# Severe hemothorax due to traumatic fracture of thoracic vertebra

**DOI:** 10.1186/s40792-024-01819-8

**Published:** 2024-01-24

**Authors:** Keigo Ozono, Kiwa Son, Kenta Momii, Yoshihiro Morifuji, Naoki Ikenaga, Masafumi Nakamura

**Affiliations:** 1 Department of Surgery and Oncology, Graduate School of Medical Science, Kyushu University, Maidashi 3-1-1, Higashi-ku, Fukuoka, 812-8582 Japan; 2 Department of Emergency, Graduate School of Medical Science, Kyushu University, Fukuoka, Japan; 3 Department of Surgery, Kyushu Rosai Hospital, Fukuoka, Japan

**Keywords:** Hemothorax, Traumatic fracture, Thoracic vertebra

## Abstract

**Background:**

Hemothorax occurs in approx. 0.4% of all chest injury patients, but hemothorax due to a thoracic vertebral fracture is rare.

**Case presentation:**

A 76-year-old Japanese man was transported to our hospital for right hemothorax due to a car accident. We performed emergency hemostasis surgery and tried to stop the bleeding by several methods, but it was difficult to control the bleeding because the bleeding point was an artery branch that runs in front of the vertebral body.

**Conclusion:**

It is important to be aware that a fractured vertebra can damage the aorta's arterial branch and follow a severe course.

## Background

Hemothorax occurs in approx. 0.4% of all patients with a chest injury [1] and is usually caused by damage to the lung parenchyma, pulmonary hilum vessels, heart, major vessels, and/or intercostal vessels [2]. Hemothorax due to a thoracic vertebral fracture is rare, and there are few reports of it. We describe the details of a patient with severe hemothorax due to a traumatic fracture of a thoracic vertebra.

## Case presentation

**Fig. 1:**
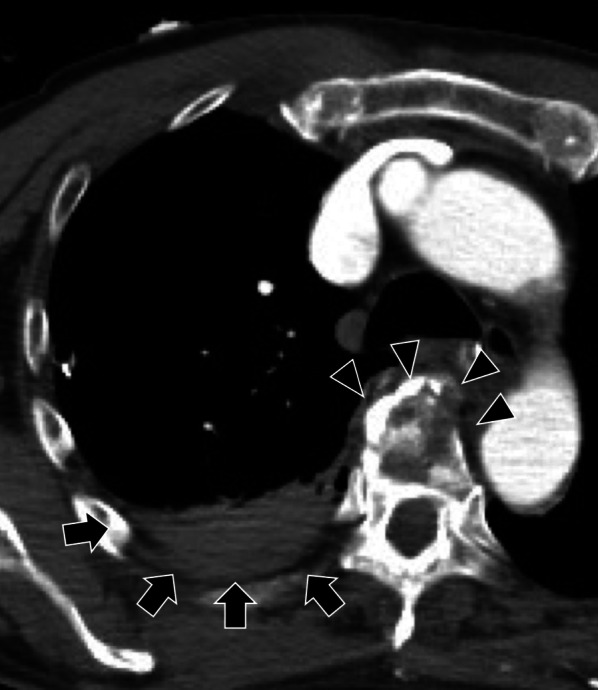
Contrast CT images showing the injuries sustained by the patient. Axial view of the chest, highlighting the fluid collection on the dorsal side of the chest cavity (*arrows*) and demonstrating the spinal body fracture of the fifth thoracic vertebra (*arrowheads*). No obvious extravasation is seen

A 76-year-old Japanese man was transported to our hospital's emergency room after a car-to-car accident. His vital signs on arrival were stable (Glasgow Coma Scale: E4V5M6, blood pressure 111/83 mmHg, heart rate 92/min, respiratory rate 18/min), but the attenuation of breath sounds of the right chest and an open fracture of the patient's forearm were observed. Ultrasonography showed fluid collection in the right chest cavity and suspected hemothorax. Chest drainage was done and approx. 800 mL bloody pleural effusion was observed, showing no continuous discharge. Contrast computed tomography (CT) showed a fracture of the first rib (precordial side) and a spinal body fracture of the fifth thoracic vertebra plus fluid collection at the dorsal side of chest cavity, without obvious extravasation (Fig. 1).

A complex fracture of the distal radius diaphysis was also seen. Temporary external-fixation fracture treatment for the radius open fracture was started. In the middle of the procedure, a drop in the patient's blood pressure and continuous blood discharge in the chest drainage tube were observed. Surgeons (we) were summoned, and when we reached the operation room, anesthesiologists were pumping the red cell concentrate into the patient. Based on the CT findings, we suspected that the bleeding was from the fractured fifth thoracic vertebra, and it was apparent that surgery performed by orthopedic surgeons was desirable. However, our hospital's orthopedic surgeons felt that the surgery would be difficult because the patient could not be placed in the prone position, due to his unstable vital signs.

**Fig. 2:**
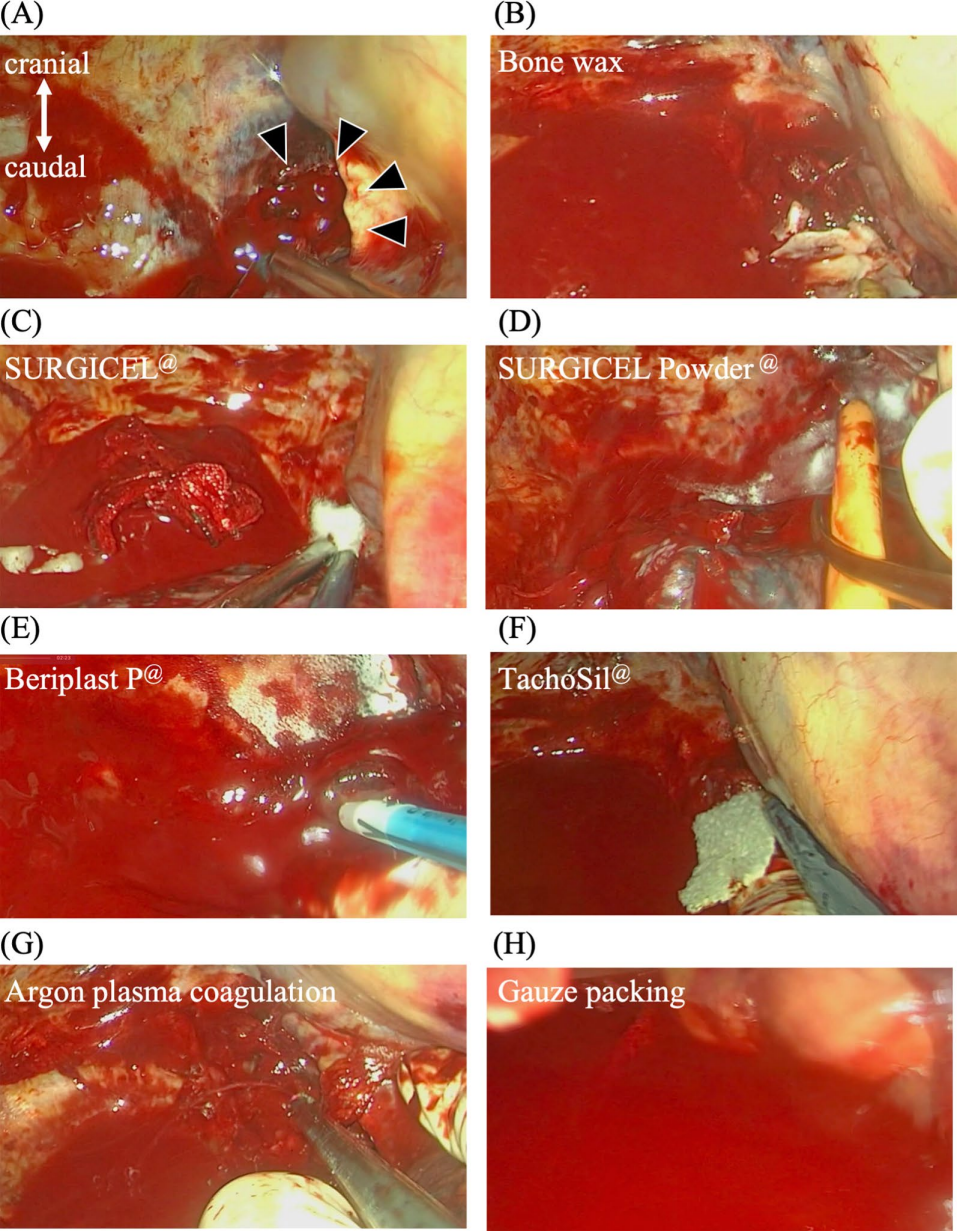

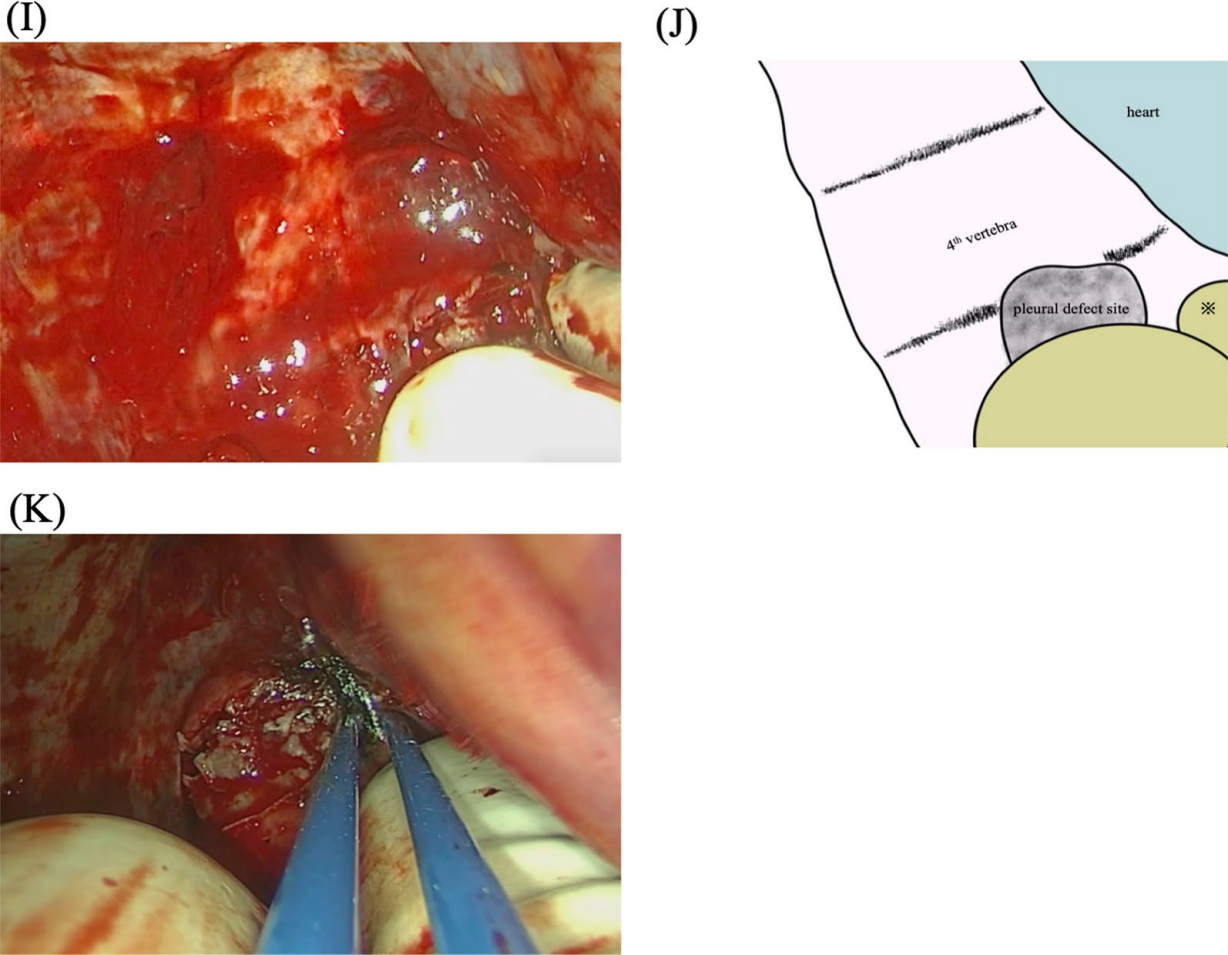
**A** During the surgery, active arterial bleeding from the fractured fifth vertebral body was observed (*arrowhead*). Various methods were applied to control the bleeding, including the use of **B** bone wax, **C** oxidized cellulose (SURGICEL), **D** powder absorbable hemostat (SURGICEL Powder), **E** a biological tissue adhesive (Beriplast P), **F** sheet-shaped biological tissue adhesive/closure (TachoSil), **G** argon plasma coagulation (APC), and **H** gauze packing. **I** We discovered that the bleeding could be controlled by pressing the mediastinal pleura above the bleeding. **J** Scheme of Fig. 2-I ※ indicates the pleura directly above the bleeding point. **K** We achieved hemostasis using bipolar cautery to coagulate the pleura above the bleeding point

We then asked our hospital's radiologists whether interventional radiology (IVR) was applicable, and they noted that IVR was not suitable because there was extensive bleeding from the fractured vertebral body. Eventually, we started surgery for emergency hemostasis by performing an anterior thoracotomy through the fifth intercostal space with the patient in the supine position. As suspected in light of the CT findings, bleeding was observed from the fractured fifth vertebral body (Fig. 2A). We attempted to stop bleeding using sequentially bone wax, oxidized cellulose (SURGICEL®), powder absorbable hemostat (SURGICEL Powder®), biological tissue adhesive (Beriplast® P), sheet-shaped biological tissue adhesive/closure (TachoSil®), and argon plasma coagulation (APC), but none of these were effective (Fig. 2B–G).

At that point, it was judged difficult to achieve hemostasis through surgery, we decided to adopt a second-look surgery at a later day after palliative hemostasis was achieved by gauze packing and transfusion fresh frozen plasma. However, the gauze-packing method was not effective, either (Fig. 2H). While we were searching for other hemostasis methods, we discovered that the patient's bleeding could be controlled by pressing the mediastinal pleura above the bleeding (Fig. 2I). We finally achieved hemostasis using bipolar cautery to coagulate the pleura above the bleeding point (Fig. 2J). We filled the bleeding point with bone wax and finished the operation. The surgery's total bleeding was 12,913 mL, and the transfusion was 21,860 mL.

On postoperative day (POD) 3, an orthopedic surgeon performed posterior lumbar interbody fusion and transforaminal lumbar interbody fusion. On POD 9, the patient was weaned off the respirator, and on POD 51 he was transferred to a rehabilitation facility, with no subsequent complications.

## Discussion and conclusions

**Fig. 3:**
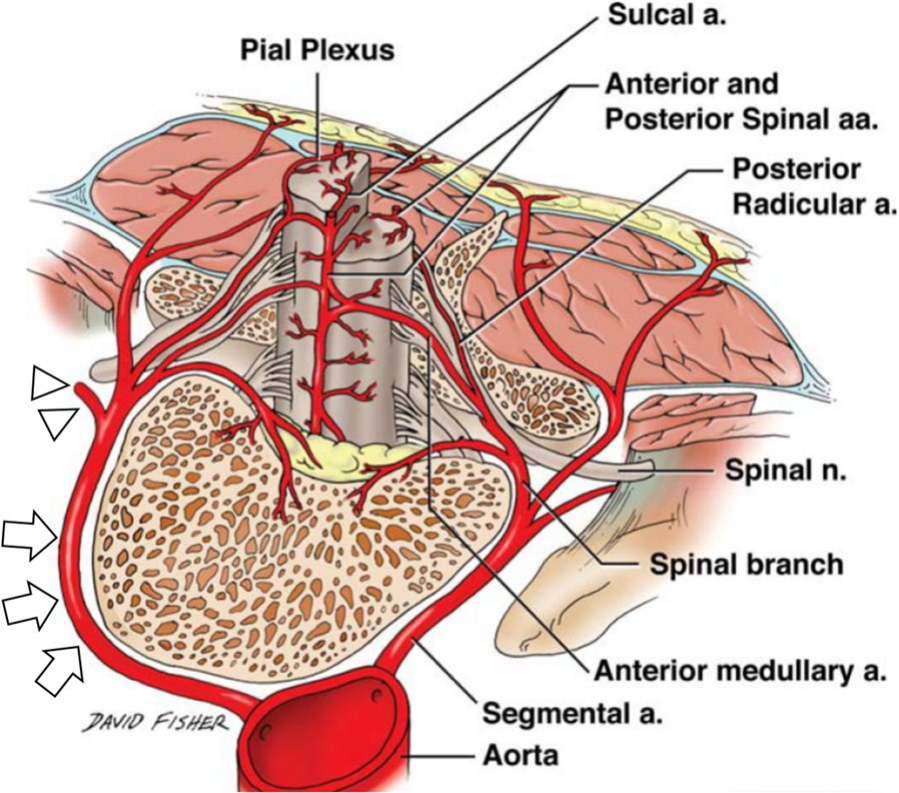
Adopted from Bosmia et al. [13]. We suspect that the bleeding was from the artery branch running in front of the vertebral body (*arrows*) and becoming intercostal arteries (*arrowheads*) after supplying the vertebral bodies

Several research groups have reported hemothorax caused by a vertebral fracture (Table 1). As shown in the table, 4 of the 13 reported patients (30.7%) died, highlighting the fact that hemothorax due to a fractured vertebral body can be fatal. Based on our review of the work published by Bosmia et al. [13], we speculated that the bleeding in our patient did not originate from the vertebral body itself but rather from the lumbar segmental artery, which originates from the aorta and primarily distributes on the lateral surface of the vertebrae (Fig. 3). These arteries give rise to intercostal branch, muscular branch, and spinal artery near the neural foramen. These are well-known bleeding points during percutaneous endoscopic lumbar discectomy in the orthopedic field [14].

**Table 1:** Reports of traumatic hemothorax by vertebral fracture

Author (year)	Age/sex	Mechanism of injury	Injury site	Intervention	Bleeding (mL)	Hemostasis method	Outcome
Dalvie (2000) [3]	28/M	Traffic accident	T4	Right thoracotomy	Unclear	Spinal fixation	Survived
Van Raaiji (2000) [4]	55/F	Fall	T11	Right thoracotomy	1500	Bone wax, synthetic patch	Survived
Kaneko (2000) [5]	86/F	Unclear	T6	Right thoracotomy	2000	Argon plasma coagulation, muscle flap	Dead
Lu (2010) [6]	72/F	Traffic accident	T11	Right thoracotomy	1300	Bone wax, gauze packing	Survived
Masteller (2012) [7]	93/M	Fall	T10 + T11	Thoracentesis	1000	Not done	Dead
Masteller (2012) [7]	71M	Fall	T11	Thoracentesis	3000	Not done	Dead
Matsushita (2016) [8]	67/M	Trauma	T3	Right thoracotomy	2090	Gauze packing, coagulant sheet	Survived
Haruta (2016) [9]	78/F	Traffic accident	T8	Left thoracotomy + clamshell	1400	Gauze packing, coagulant sheet	Dead
Okamoto (2018) [10]	81/M	Fall	T7	Right thoracotomy	1330	Bone wax, coagulant Sheet	Survived
Hirota (2019) [11]	74/F	Fall	T11	Right thoracotomy	1200	Coagulant Sheet	Survived
Ninomiya (2020) [12]	81/M	Traffic accident	T8	Right thoracotomy	1500	Gauze packing	Survived
Ninomiya (2020) [12]	64/M	Fall	T7	Right thoracotomy	1300	Gauze packing, coagulant sheet, bone wax	Survived
Our case	76/M	Traffic accident	T4	Right anterior thoracotomy	12,913	Coagulation by bipolar	Survived

In this case, since the bleeding originated near the fifth thoracic vertebra, consideration for injury to the Adamkiewicz artery was not necessary. However, if the damage site had been in the lower spine, it is necessary to provide adequate informed consent regarding the potential for postoperative complications, including paraplegia, in exchange for life-saving measures.

The bleeding point was located below our patient's pleura, and we were unable to ligate the vessel. We therefore attempted the several above-described other hemostatic methods, but the bleeding was arterial in nature and we were unable to even reduce the bleeding flow, let alone achieve hemostasis. This is why our patient had so much bleeding and to control bleeding more efficiently, making a vertical incision along the rib cartilage toward the cranial direction from the fifth intercostal incision, and performing a larger thoracotomy might have facilitated the surgical procedure. Furthermore, once it was determined that the bleeding was pinpoint rather than extensive from the fractured vertebral body, it might have been possible to considered IVR.

Fortunately, we discovered that applying pressure to the pleura at the bleeding point could control the bleeding. We then used bipolar cautery to coagulate the affected area of the pleura from above and thus achieve hemostasis.

This is apparently the only published report of an anterior thoracotomy performed with the patient in a supine position for hemothorax caused by a vertebral fracture, and it illustrates that it is important to be aware that a fractured vertebra can injure the lumbar segmental arteries and follow a severe course.

## Data Availability

The data in this report can be obtained from the corresponding author on reasonable request.

## Abbreviations

CT: Computed tomography
IVR: Interventional radiology
APC: Argon plasma coagulation
POD: Postoperative day
